# A computational approach based on weighted gene co-expression network analysis for biomarkers analysis of Parkinson’s disease and construction of diagnostic model

**DOI:** 10.3389/fncom.2022.1095676

**Published:** 2023-01-10

**Authors:** Zhaoping Wu, Zhiping Hu, Yunchun Gao, Yuechong Xia, Xiaobo Zhang, Zheng Jiang

**Affiliations:** ^1^Department of Neurology, The Second Xiangya Hospital, Central South University, Changsha, Hunan, China; ^2^Department of Neurology, The First People’s Hospital of Changde City, Changde, Hunan, China; ^3^Department of Respiratory Medicine, Central South University, Changsha, Hunan, China

**Keywords:** Parkinson’s disease, WGCNA, LASSO regression, potential diagnostic predictor, computational approach

## Abstract

**Background:**

Parkinson’s disease (PD) is a common age-related chronic neurodegenerative disease. There is currently no affordable, effective, and less invasive test for PD diagnosis. Metabolite profiling in blood and blood-based gene transcripts is thought to be an ideal method for diagnosing PD.

**Aim:**

In this study, the objective is to identify the potential diagnostic biomarkers of PD by analyzing microarray gene expression data of samples from PD patients.

**Methods:**

A computational approach, namely, Weighted Gene Co-expression Network Analysis (WGCNA) was used to construct co-expression gene networks and identify the key modules that were highly correlated with PD from the GSE99039 dataset. The Least Absolute Shrinkage and Selection Operator (LASSO) regression analysis was performed to identify the hub genes in the key modules with strong association with PD. The selected hub genes were then used to construct a diagnostic model based on logistic regression analysis, and the Receiver Operating Characteristic (ROC) curves were used to evaluate the efficacy of the model using the GSE99039 dataset. Finally, Reverse Transcription-Polymerase Chain Reaction (RT-PCR) was used to validate the hub genes.

**Results:**

WGCNA identified two key modules associated with inflammation and immune response. Seven hub genes, LILRB1, LSP1, SIPA1, SLC15A3, MBOAT7, RNF24, and TLE3 were identified from the two modules and used to construct diagnostic models. ROC analysis showed that the diagnostic model had a good diagnostic performance for PD in the training and testing datasets. Results of the RT-PCR experiments showed that there were significant differences in the mRNA expression of LILRB1, LSP1, and MBOAT7 among the seven hub genes.

**Conclusion:**

The 7-gene panel (LILRB1, LSP1, SIPA1, SLC15A3, MBOAT7, RNF24, and TLE3) will serve as a potential diagnostic signature for PD.

## 1. Introduction

Parkinson’s disease (PD) is an age-related disease and is the second most common chronic neurodegenerative disorder (Dassati et al., 2021). Its clinical symptoms include static tremors, muscle tension, and loss of smell (Bostantjopoulou et al., 2013). Although Positron Emission Computed Tomography (PET) imaging of dopamine transporter is used in the diagnosis of PD, it is expensive and inconvenient and therefore not suitable for some medical centers (Nicastro et al., 2021). Currently, PD is mainly diagnosed based on physical examination and clinical symptoms, and there are no affordable, effective, and less invasive examination methods (Farotti et al., 2020).

Biomarkers in peripheral blood samples are a good solution to the diagnosis of PD. Early α-synuclein and DJ-1 are considered as possible peripheral biomarkers, but experimental results show that they are not ideal (Chahine et al., 2014). Later studies found that low levels of peripheral uric acid (Weisskopf et al., 2007), epidermal growth factor (Chen-Plotkin et al., 2011), and apolipoprotein A1 protein predicted an increased risk of PD (Qiang et al., 2013). In recent years, with the development of bioinformatics, gene expression microarray, or next generation sequencing technology is an ideal method to screen PD biomarkers from peripheral blood transcripts (Shamir et al., 2017; Falchetti et al., 2020; Jin et al., 2020; Lin et al., 2021). In addition to transcriptomics, epigenetics, and proteomics are also good ways to explore potential biomarkers for the diagnosis of PD (Mayo et al., 2021).

The difficulty in the diagnosis of PD lies not only in the location of the lesion in the central nervous system, where most biological factors are sequestered by the blood-brain barrier, resulting in fewer biomarkers in peripheral blood, but also that the clinical manifestations of PD overlap with other neurodegenerative diseases. In this study, we used bioinformatics methods to screen for potential diagnostic markers of PD in peripheral blood from Gene Expression Omnibus (GEO) datasets in samples containing PD and other neurodegenerative diseases (NDD).

## 2. Materials and methods

### 2.1. Microarray data preprocessing

The data of GSE99039 analyzed during the current study are available from the GEO database. The GSE99039 is the largest sample size dataset of all PD peripheral blood transcriptional analysis datasets and contained 205 idiopathic PD patients, 233 healthy controls, and 48 NDD patients [including 27 patients with Huntington disease, and 21 with Multiple System Atrophy (MSA), Corticobasal Degeneration (CBD), progressive supranuclear palsy (PSP), or PD dementia (PDD)] (Shamir et al., 2017). These data were generated using the GPL570 platform (Affymetrix Human Genome U133 Plus 2.0 Array).

The raw gene expression data and clinical trait data of GSE99039 were downloaded from the GEO database. The Affy package in R was used for background correction and quantile normalization of the raw expression data (Gautier et al., 2004), whereas the ArrayQualityMetrics package was used for quality assessment to remove unqualified samples (Distances between arrays, Boxplots, MA plots, two of the three items are unqualified as unqualified sample: 1 PD, 1 NDD, and 3 control) (Kauffmann et al., 2009). The SVA package in R was used to remove batch effects. Platform annotation files were used to annotate the probes, and the average expression levels of genes represented by more than one probe were calculated (Leek et al., 2012). The GSE99039 dataset was randomly divided into the training set and test set at a ratio of 7:3 (Table 1).

**TABLE 1:** Samples of training, testing, and validation datasets after preprocessing.

	Training	Testing
PD	156	48
NDD	41	6
Normal	157	73
Total	354	127

### 2.2. Construction of co-expression network with WGCNA

WGCNA package in R was used to construct the co-expression network and identify co-expression gene modules. First, genes with the top 25% variance in gene expression from the GSE99039 training set were used to construct scale-free co-expression networks. To construct scale-free networks, we chose the soft threshold power value. Subsequently, we used Pearson correlation matrices to calculate a correlation matrix for the genes. Next, we transformed the correlation matrix into a weighted adjacency matrix using the power function. Finally, we performed step-by-step network construction and module detection using the following major parameters: deepSplit = 2, power = 11, networkType = unsigned, minModuleSize = 30, and mergeCutHeight = 0.20. We identified the key modules that are strongly associated with PD based on the correlation between modules and disease group (Langfelder and Horvath, 2008).

### 2.3. Gene ontology and KEGG enrichment analysis

ClusterProfiler package was used to perform Gene Ontology (GO) and Kyoto Encyclopedia of Genes and Genomes (KEGG) enrichment analysis of genes in key modules (Yu et al., 2012).

### 2.4. Identification of hub genes and construction a prediction gene signature

Module membership (MM) represents the intramodular connectivity of any gene in a given module. A higher absolute value of MM indicates that a gene has a higher negative or positive correlation with the module eigengenes (MEs). Gene significance (GS) is used to incorporate external information into the co-expression network, with a higher absolute value of GS indicating the increased biological significance of a gene for a given clinical trait. We selected hub genes in key modules based on the criteria: | MM| > 0.8 and | GS| > 0.2 (Xia et al., 2020). We performed the LASSO regression analysis to extract genes strongly associated with PD in the training dataset *via* glmnet package in R.

### 2.5. Construction of the diagnostic model

The selected genes were used to construct a diagnosis model based on multivariate Logistic regression in the training set in the glmnet package in R. To evaluate the ability of the Logistic regression model to identify PD, Receiver Operating Characteristic (ROC) curve analysis conducted by the pROC package and confusion matrix were used in the training set and test set (Robin et al., 2011).

### 2.6. RT-PCR validation of the hub genes

Serum samples from five patients without PD and five patients with PD were collected for Reverse Transcription-Polymerase Chain Reaction (RT-PCR) validation to validate the predictive power of the signature. This protocol was approved by the Ethics Committee or Institutional Review Board of Changde First People’s Hospital (Approval number: 2018-028-01). Total RNA samples were extracted using the TRIzol reagent (Thermo, 15596026) according to the manufacturer’s instructions. The RNA was reverse-transcribed to cDNA which was prepared using the SYBR qPCR reaction system (Thermo, PIKOREAL96, USA) for RT-PCR. Glyceraldehyde-3-phosphate dehydrogenase (GAPDH) served as the internal control. Relative mRNA expression was calculated using the ΔΔCT method. Primers used are shown in Table 2.

**TABLE 2:** Primer information.

Target name	Primer
GAPDH	ACAGCCTCAAGATCATCAGC
	GGTCATGAGTCCTTCCACGAT
LILRB1	GCACCCTGGATTACACGGAT
	GATACCGCCCTGTGTGTTCCC
LSP1	GGTTCAGGCTTCAGTCCCAG
	CCTCCCCTTTACGGTTCCAG
SIPA1	CCACAGCCAAGCCATCAGTA
	TTCCGCAGAGACAAGGTACG
SLC15A3	CCAAGGACTTTGGGAACATCA
	CTCTCATAGCGTCCAGCGAT
MBOAT7	ACTATGAGACCATCCGCAAC
	ACTGCACCGTCATGTTCCA
RNF24	TCCAGAATCTGCCTCTCAACA
	ATTCTTTGTGTGCTTGATGTCT
TLE3	CCACCATGAGATGAACGGCT
	AACAGCTCCAAAGCTCACCA

### 2.7. Statistical analysis

*T*-test was used for RT-PCR data analysis. All statistical analysis was performed in the R software (v. 4.1.2). Unless otherwise stipulated, *P* < 0.05 was regarded as statistically significant.

## 3. Results

### 3.1. Weighted gene co-expression network analysis

**FIGURE 1:**
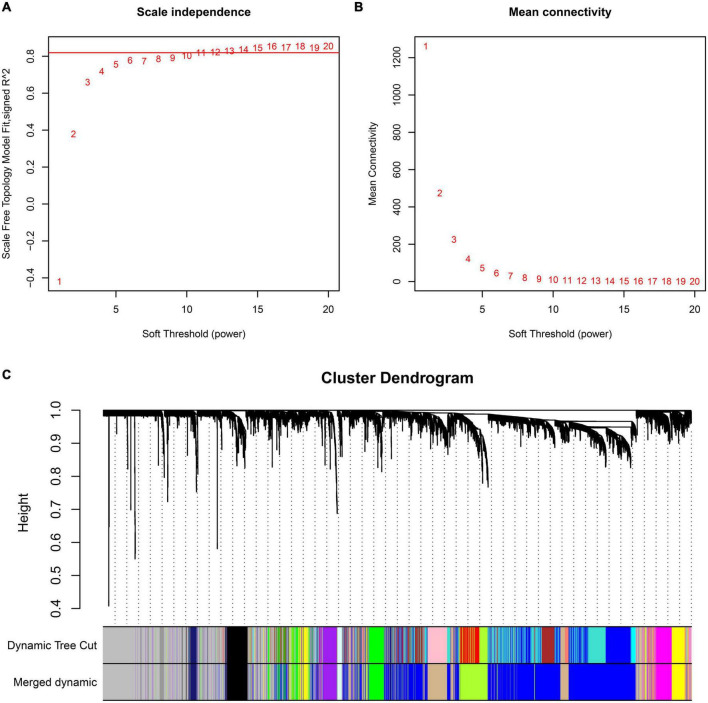
Construction of WGCNA co-expression modules. Analysis of the scale-free fit index **(A)** and the mean connectivity **(B)** for various soft-thresholding powers. **(C)** Dendrogram of all differentially expressed genes clustered based on a dissimilarity measure (1-TOM).

Preprocessing of the GSE99039 training dataset resulted in the identification of 21,655 genes expression profiles. Based on genes with the top 25% variance in expression, 5,414 genes were selected for WGCNA. To construct an approximate scale-free topological network, we choose the power 11, which is the lowest power for the scale-free topological fitting exponential curve to flatten out when its value goes beyond 0.8 (Figure 1A) and mean connectivity is close to 0 (Figure 1B). The identified genes were divided into 12 modules, with the gray module consisting of genes that could not be clustered (Figure 1C).

**FIGURE 2:**
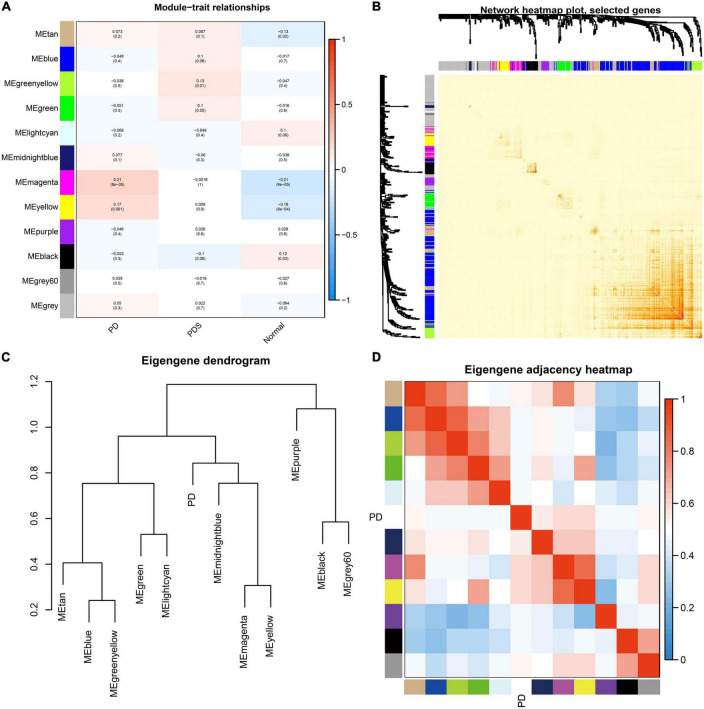
Correlation analysis of modules from weighted gene co-expression network analysis and identification of PD-associated modules. **(A)** Heatmap of correlation between clinical traits (*x*-axis) and modules (*y*-axis). The abscissa represents the clinical information (PD, NDD, Normal) contained in the dataset, and the ordinate represents different modules. The histogram on the right is the color scale. Numbers inside the heatmap signify correlation values and *p*-values (in parenthesis). **(B)** Heatmap depicting the interaction of co-expressed genes. Different colors in both axes represent different modules, and the brightness of red in the middle of heatmap indicates the connectivity degree of the corresponding modules. **(C)** Hierarchical clustering dendrogram displaying the similarity of each module eigengenes value. **(D)** Heatmap showing correlation between each module, labeled by their corresponding color.

The magenta and yellow modules had the highest correlation with the PD group (*r* = 0.21, *p* < 0.01; *r* = 0.17, *p* < 0.01) (Figure 2A), and were thus identified as key modules. The interaction relationships among the 11 modules obtained from WGCNA were plotted as network heatmap (Figure 2B). The results showed a high degree of independence between these modules, which means that gene expression among the modules is relatively independent. In addition, to represent the similarity of modules, we performed hierarchical clustering according to the signature gene values of each module (Figure 2C). The results showed that these modules were mainly divided into three clusters, and the results of the module-associated heatmap were consistent (Figure 2D).

### 3.2. GO and KEGG enrichment analysis

Gene Ontology and KEGG enrichment analysis was performed on 210 genes in the magenta module and 272 genes in the yellow module. The GO analysis was performed in relation to three aspects: biological process, cellular component, and molecular function.

**FIGURE 3:**
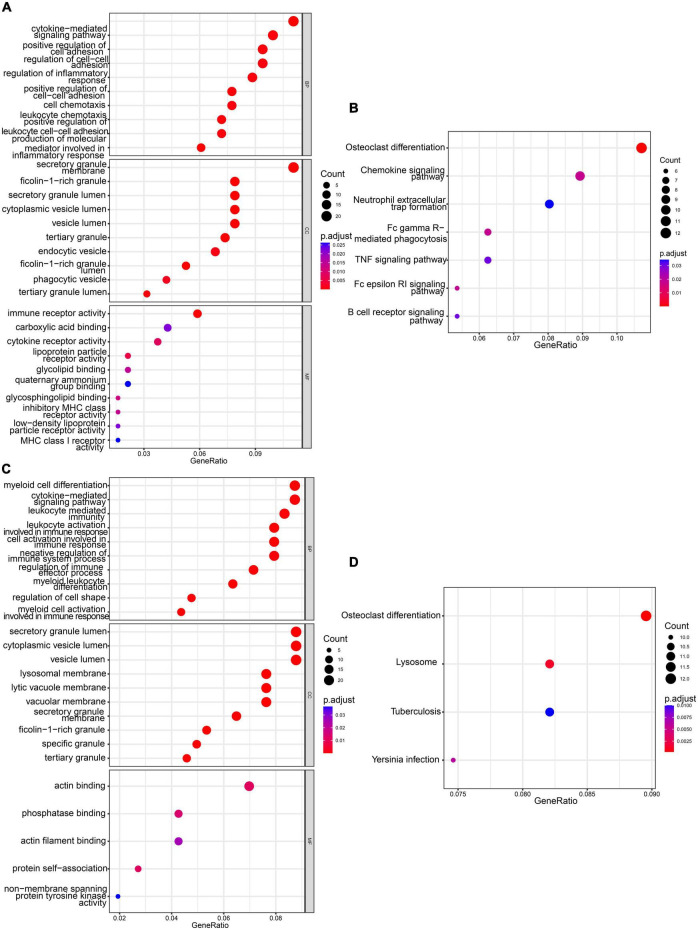
Functional enrichment analyses of magenta and yellow modules. **(A)** GO analysis of genes in magenta module. **(B)** KEGG pathway analysis of genes in magenta module. **(C)** GO analysis of genes in yellow module. **(D)** KEGG pathway analysis of genes in yellow module.

Magenta module GO terms were enriched in “cytokine-mediated signaling pathway,” “secretory granule membrane,” and “immune receptor activity” (Figure 3A). The KEGG pathway analysis results showed that the genes were enriched in pathways involved in “Osteoclast differentiation” and “Chemokine signaling pathway” in the magenta module (Figure 3B).

Yellow module GO terms were enriched in “myeloid cell differentiation,” “secretory granule lumen,” and “actin binding” (Figure 3C). The KEGG pathway analysis results showed that the genes were enriched in “Osteoclast differentiation” and “lysosomal pathways” (Figure 3D).

Results from GO and KEGG enrichment analysis indicated that the genes in the key modules may be involved in the development of PD.

### 3.2. Selection of potential biomarkers for PD diagnosis

**FIGURE 4:**
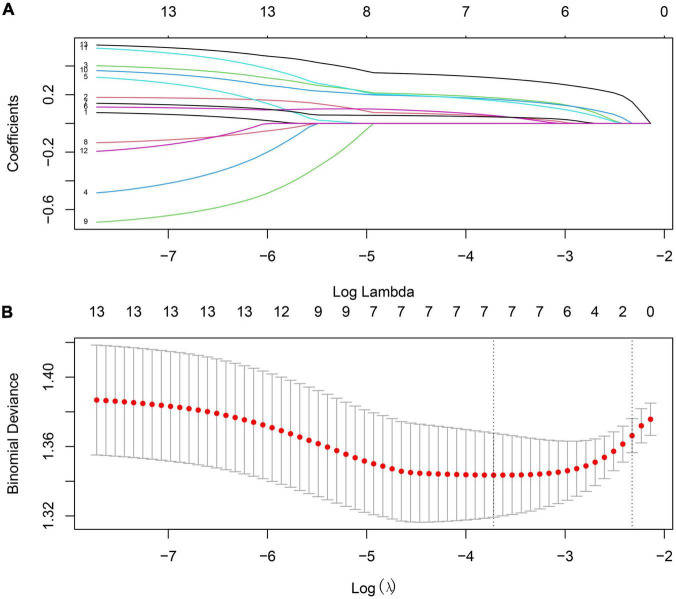
The process of screening genes most associated with prognosis in the training set by Lasso regression. **(A)** Processes of LASSO regression model fitting. **(B)** The misclassification error in the jackknife rates analysis.

Based on the criterion: | MM| > 0.8 and | GS| > 0.2, we identified seven hub genes from the magenta and yellow modules, respectively (Table 3). Next, we performed the Least Absolute Shrinkage and Selection Operator (LASSO) regression analysis to identify the relationship between genes in key modules and the PD patients and using the glmnet package in R (Figure 4). Consequently, seven genes, LILRB1, LSP1, SIPA1, SLC15A3, MBOAT7, RNF24, and TLE3 were found to be highly associated with PD in the GSE99039 training dataset.

**TABLE 3:** Magenta and yellow modules Hub gene’s MM and GS.

Modules	Gene symbol	MM	GS
Magenta	DGAT2	0.890622815	0.200158516
	HCK	0.856668415	0.201961097
	MBOAT7	0.841048131	0.226820797
	RNF24	0.830176878	0.220654612
	FOSL2	0.817954018	0.202700685
	GPR97	0.837251440	0.209726069
	TLE3	0.879416377	0.237222540
Yellow	C15orf39	0.802615634	0.208988011
	LILRB1	0.833006299	0.204477963
	LSP1	0.834070703	0.217503094
	RHOG	0.859659866	0.202549826
	RPS6KA1	0.865249043	0.211739457
	SIPA1	0.862102043	0.201769263
	SLC15A3	0.835962872	0.213660706

### 3.3. Construction of gene diagnosis model

The seven genes were used to construct a diagnosis model using multivariate Logistic regression. The gene-based model index was created according to the following formula: index = (1.09 × expression of LILRB1) + (1.26 × expression of LSP1) + (1.13 × expression of SIPA1) + (1.06 × expression of SLC15A3) + (1.24 × expression of MBOAT7) + (1.25 × expression of RNF24) + (1.45 × expression of TLE3).

**FIGURE 5:**
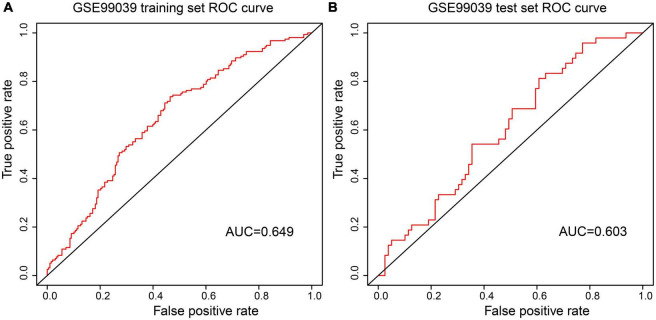
ROC curves were used to evaluate the accuracy of logistic regression model. **(A)** Analysis of the GES9903 training set. **(B)** Analysis of the GES9903 test set.

Receiver Operating Characteristic curve analysis indicated that the AUC of the 7-gene-based model was 0.65 in the training set and 0.60 in the test set (Figures 5A, B). The confusion matrix results show that the False Positive Rate (FPR), False Negative Rate (FNR), and Error Rate (ER) of the model in the training set are 0.263, 0.545, 0.387, respectively, and 0.241, 0.667, 0.402 in the test set respectively (Tables 4, 5). These suggests that the Logistic regression model had stability.

**TABLE 4:** Training set confusion matrix.

Total = 354		Actual	
		Control	PD
Predicted	Control	146	85
	PD	52	71

**TABLE 5:** Test set confusion matrix.

Total = 127		Actual	
		Control	PD
Predicted	Control	60	32
	PD	19	16

### 3.4. The experiment of seven hub genes

**FIGURE 6:**
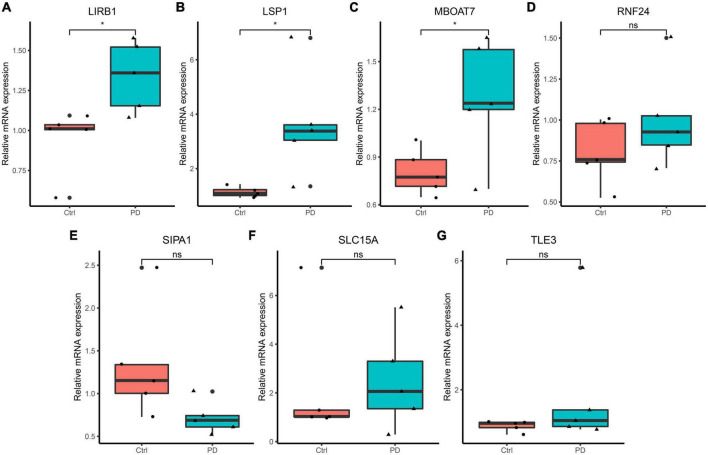
Reverse Transcription-Polymerase Chain Reaction validation of the hub gene between PD and normal controls. **(A–G)** Relative expression levels of LILRB1, LSP1, MBOAT7, RNF24, SIPA1, SLC15A3, and TLE3. *p < 0.05.

Reverse Transcription-Polymerase Chain Reaction results of RNAs isolated from peripheral blood samples of PD patients showed that mRNA expression levels of LSP1, LIRB1, and MBOAT7 in the PD group were higher than those in the control group (< 0.05), but the expression levels of SIPA1, SLC15A3, TLE3, and RNF24 was not significantly different between the two groups (Figure 6). The identified seven genes may serve as potential diagnostic biomarkers.

## 4. Discussion

Parkinson’s disease is a type of NDD commonly seen in the elderly. The main motor symptoms of this disorder are caused by the degeneration of dopamine neurons in the substantia nigra that innervate the striatum (Pajares et al., 2020). Currently, the diagnosis of PD is mainly based on clinical examination. Therefore, there is need to develop a more accurate, widely applicable, and specific diagnostic technique for PD.

Using WGCNA, we identified two key modules that were able to distinguish patients with PD from other NDD patients and healthy individuals. Then we used seven hub genes LSP1, LIRB1, MBOAT7, SIPA1, SLC15A3, TLE3, and RNF24 from the key modules to construct a PD prediction model. GO and KEGG enrichment analysis showed that the genes in the two Hub modules were mainly related to inflammation and immunity. The seven genes in the model, all associated with inflammation or immunity, were highly expressed in the bone marrow, lymph nodes, or brain. Studies have demonstrated that inflammation is an important factor in the development of PD, but the exact underlying mechanism is unclear. The innate immune mechanism of PD patients is dysregulated, microglial cell proliferation can be seen in the brain (McGeer et al., 1988), and there are autoantibodies against α-synuclein protein, dopamine, and melanin in serum and cerebrospinal fluid (Double et al., 2009; Yanamandra et al., 2011), suggesting that humoral immunity may play a role in PD-related neuroinflammation and neurodegeneration. In addition to microglia and astrocyte hyperplasia in the brain of PD patients, peripheral inflammation, and PD risk-related genes can also promote chronic inflammatory responses that lead to the progression of this neurodegenerative disease (Pajares et al., 2020), and cytokine such as IL1β, IL2, IL6, IFN-γ, and TNF-α can be detected in peripheral blood (Reale et al., 2009).

The LSP1 gene encodes an intracellular F-actin binding protein expressed in lymphocytes, neutrophils, macrophages, and endothelial cells (Palker et al., 1998). The protein can regulate the movement of neutrophils, adhesion to fibrinogen matrix proteins, and *trans*-endothelial migration. It is also expressed in CD8 + T cells and is associated with T-cell maturation, and chemotaxis of neutrophils (Wu et al., 2007). CD8 + T cells play an important role in PD development. In the early stages of PD, infiltration of the substantia nigra of CD8 + T cells precedes synuclein deposition. The infiltration of cytotoxic CD8 + T cell in the substantia nigra takes place before α-synuclein aggregation and neuronal death, and it parallels the progression of neuronal death and synuclein disease in PD (Galiano-Landeira et al., 2020). Oxidative modification of specific proteins associated with PD (i.e., α-syn nitrification) has been implicated in the generation of new epitopes that initiate peripheral blood-driven CD4 + and CD8 + T cell responses (Benner et al., 2008). CD8 (+) T-cells are mainly expressed in the peripheral immune system of PD patients, while the levels of CD4 (+) CD25 (+) T-cells tend to be low (Baba et al., 2005). The LILRB1 gene is a member of the leukocyte immunoglobulin-like receptor (LIR) family, which is present in the gene cluster 19Q13.4 in the chromosome (Truong et al., 2019). The receptor is expressed on immune cells, binds to MHC class I molecules on antigen-presenting cells, and transduces negative signals that inhibit stimulation of immune response. Activation of LIR suppresses NKR expression in late differentiation of CD8 + T cells (Martínez-Rodríguez et al., 2010).

MBOAT7 encodes a membrane-binding protein, lysophosphatidylinositol transferase 1 (LPIAT1), which has 472 amino acids and is present in the inner membranes of organelles such as endoplasmic reticulum and mitochondria (Gijón et al., 2008). It is mainly involved in the metabolism of phospholipids, and is not directly involved in the oxidative energy pathway. MBOAT7 regulates free arachidonic acid (AA) in cells by remodeling phospholipids (Dursun et al., 2020). Free AA is up-regulated in the brain of PD animal models, and metabolism-related COX-2 of AA increases (Lee et al., 2010). LPIAT1 regulates the levels of arachidonic acid in phosphatidylinositol, which is necessary for cortical lamination in mice (Lee et al., 2012). MBOAT7 gene mutation or abnormal expression has been associated with mental impairment, epilepsy (Jacher et al., 2019), and changes in MRI signals of the cerebral pallidus (Ozpinar et al., 2021).

The TLE3 gene encodes transcriptional co-repressors belonging to the transduction protein-like enhancer family (Dehni et al., 1995). Members of this family play a role in the Notch signaling pathway, and TLE3 expression is associated with neurogenesis and epithelial differentiation. Inhibition of the Notch signaling pathway is known to alleviate PD symptoms (Wang et al., 2019). Interestingly, DNER-an activator of the NOTCH1 pathway was higher in PD than in atypical parkinsonisms (Santaella et al., 2020). TLE3 is involved in immune regulation, promotes memory B-cell development (Ascoli et al., 2022), and acts as a predictor of peripheral CD4 + T cell depletion (Laidlaw et al., 2020).

The product of SIPA1 gene is the mitogen-induced GTP-activating protein (GAP), which is located in the perinuclear region and affects mitogen-induced cell cycle progression (Wang et al., 2020). It is associated with lymphocyte proliferation (Minato, 1996) and regulation of T-cell function (Ishida et al., 2003). SLC15A3 contributes to the dipeptide transmembrane transporter, participates in dipeptide input across the plasma membrane, and is located in the inner membranes of the cell organelles (He et al., 2018). Studies have shown that SLC15A3 is regulated by various TLRS and plays an important role in regulating TLR4-mediated inflammatory response (Song et al., 2018). RNF24 gene encodes a complete membrane protein containing ring zinc finger. The encoded proteins may interact with multiple transient receptor potential cationic channel subfamily C (TRPC) proteins and regulate their transport and insertion into the plasma membrane (Lussier et al., 2008). RNF24 gene is associated with viral immunity (Samus et al., 2020).

LSP1 is the one with the highest efficacy value among the differentially expressed genes verified by RT-PCR in the model, and its biological function is consistent with the peripheral inflammation of PD, so it’s considered a critical gene among the seven hub genes. We believe that GO and KEGG pathways implicated by models, the more genes are enriched, the more important they are for PD. Therefore, three GO terms and one KEGG pathway are important for PD in our model. They were myeloid cell differentiation, cytokine mediated signaling pathway, actin binding, and osteoclast differentiation. Among them, studies on osteoclasts and PD have increased in recent years (Malik et al., 2021; Hong et al., 2022), which indicates that osteoclasts may be a new research direction for Parkinson’s mechanism or peripheral biomarkers.

The limitation of this study is that the number of cases included in the verification experiment is relatively small. Increasing the number of verification experiment samples may lead to better experimental results. At the same time, there is a lack of *in vitro* experiments to study the relevant mechanism. In summary, we found seven genes that are potential biomarkers of PD.

## 5. Conclusion

Our study identified new potential diagnostic biomarkers for PD in peripheral blood and provided insights for developing effective diagnostic and therapeutic strategies for patients with PD.

## Data availability statement

The datasets presented in this study can be found in online repositories. The names of the repository/repositories and accession number(s) can be found in the article/supplementary material.

## Ethics statement

The studies involving human participants were reviewed and approved by the Ethics Committee or Institutional Review Board of Changde First People’s Hospital (Approval number: 2018-028-01). The patients/participants provided their written informed consent to participate in this study.

## Author contributions

ZW: conceptualization, methodology, visualization, software, and writing—original draft. YX: methodology. XZ: writing and editing. YG: editing and funding acquisition. ZH: supervision and funding acquisition. ZJ: validation. All authors contributed to the article and approved the submitted version.
